# Groundwater Storage Trends and Their Link to Farmer Suicides in Maharashtra State, India

**DOI:** 10.3389/fpubh.2019.00246

**Published:** 2019-08-30

**Authors:** Pennan Chinnasamy, Minna J. Hsu, Govindasamy Agoramoorthy

**Affiliations:** ^1^N. M. Sadguru Water and Development Foundation, Dahod, India; ^2^Department of Biological Sciences, National Sun Yat-sen University, Kaohsiung, Taiwan; ^3^College of Pharmacy and Health Care, Tajen University, Yanpu, Taiwan

**Keywords:** GRACE, groundwater crisis, farmer suicide, land degradation, India, Maharashtra, CGWB, social consultations

## Abstract

Threats posed by land degradation and desertification continue to destabilize India's agriculture productivity and food security. The enduring negative environmental consequences of the agricultural intensification that started during the 1960s have further contributed to the depletion of soil nutrients in farmlands through heavy input of chemical fertilizers and pesticides. More than half of India's population depends on farming. When crop productivity fails, the economically-depressed farmers are unfortunately pushed toward suicide. The news of farmers committing mass suicide in Maharashtra state of India has lately received world attention. Although suicide may involve various psychological, social and economic factors, access to irrigation water remains a contentious matter in the agriculture business. The groundwater (GW) data from government sources are limited and not displayed in the public domain for verification. Hence, this study used the Gravity Recovery and Climate Experiment (GRACE) satellite data to compare farmer suicide rate in Maharashtra with GW storage estimates at broad spatiotemporal scales. The results show significant correlations (*r* = −0.85, *p* < 0.005) between water storage and suicide rate when time lags of 2 years are introduced between them. Based on the new findings, this study recommends that the GW status needs to be monitored scientifically across India's suicide zone. By doing so, the worst case scenarios can be logically predicted well in advance. The government then may have the advantage to mobilize its resources on the ground to implement rapid emergency measures and farmer consultations to minimize future farmer suicide cases.

## Introduction

India harbors nearly 640,000 villages that are widely spread across an area of 3.3 million km^2^ supporting nearly 1.3 billion people, of which over half depend on agriculture directly and indirectly for their livelihoods (1). The farming communities largely rely on the seasonal monsoon rains and freshwater (surface/ground) resources to cultivate crops. The farming support to India's GDP has been gradually reducing from 38% in 1975 to 16% in 2014. The continuing land degradation problems and unsustainable development undertakings indeed make India's future agriculture outlook bleak [(2, 3), Agoramoorthy, 2017].

The mitigation policies imposed by the government over the last few decades have not reversed the detrimental land degradation trend in rural farmlands and over 30–40% of India's land area falls now under the category of degraded land (4, 5). Unsustainable land use patterns involving intensive farming of marginal lands, inappropriate crop rotation patterns, severe loss of natural forests influenced by shifting cultivation, aggressive land development induced by globalization, government subsidy for chemical fertilizers, declining farmland-to-manpower ratio, and improper use of irrigation water have aggravated land degradation across the country (6–8). As a matter of fact, the farmers are the most affected by land degradation financially since they are the poorest in the Indian society. Many studies have indicated that poor farmers often became the ultimate victims of land degradation in many developing nations (9, 10). As a result, land degradation and water scarcity often leads to crop loss that accelerates the suicide domino effect (11, 12).

Between 1995 and 2010, about 17,850 farmers have committed suicides in India (13–16). Studies have shown that suicides are generally concentrated in some regions (17, 18), especially in Maharashtra, Andhra Pradesh and Karnataka states (16, 19). In fact, Maharashtra state leads in the number of farmer suicide with 60,750 cases reported between 1995 and 2013 (15, 20). Furthermore, 3,685 cases were reported yearly from 2004 to 2013 in Maharashtra alone, which equals to 10 suicides per day or one every 2 h. The hardest hit region was Vidarbha where 95% of victims suffered heavy debt (19, 21). Reliable quantification of this bizarre death phenomenon is essential to take timely scientific measures to mitigate the crisis (22), however many challenges in quantification exist.

The existing cross-boundary tools are not sufficient to comprehend the complex association between water accessibility and farmer suicide. Hence, it is essential to investigate how key factors could impact farmers at the grassroots level. Of the key factors, land, water, mental health (23, 24) and food security are intricately connected to farmers (25). Although farming accounts for over 60% of water usage in India (26), most of which from using groundwater (GW) resources, past studies have not correlated GW status to suicide rates [e.g., (27)]. This study, for the first time, correlated the GW storage trends with farmer suicide rate to understand the scientific significance. In addition, time lag models were created to understand if early deduction of farmer suicide was possible. The current study used the monthly hydrological signals of Terrestrial Water Storage (TWS) from GRACE satellites, and simulated Soil Moisture (SM) variations from Global Land Data Assimilation System's (GLDAS) Noah model to depict depletion in Maharashtra's GW and compared it against farmer suicide rates.

GRACE data were used successfully in the past to determine the GW status to develop effective water resource management plans worldwide (28–32). This study has been designed to comprehend the correlation between farmer suicide rates and depletion of GW level between 2002 and 2013 in one of India's largest states, Maharashtra. Normally correlations between two variables are performed holding the time constant between them (i.e., on same timestamps), however, this study also investigated lagged timestamps in groundwater vs. farmer suicides, wherein the timeframe is not held constant This was necessary since the monsoon, which is the major sources of groundwater recharge, arrives in the second half of the year. The recharged groundwater is therefore mostly useful for the following years, and not the same calendar year. Such a correlation with lag time will contribute to the development of better land and water resource management measures aimed at reducing farmer suicide scenarios before the disaster. Statistical models were used to establish correlations between suicide rates and remotely-sensed water storage data. Time lags have been introduced for the water storage data to distinguish the influence of water storage status on farmers' suicide rates.

## Objectives

The major aim of this study is to investigate the relationship between groundwater availability and farmer suicide in India. Of the physical factors that promote farming, since water availability is one of the most important factors, the primary objective focuses on this physical factor. Secondary objectives include the investigation of time-lagged relationship between groundwater availability and farmer suicide and statistical analysis of prediction models.

## Methods

The Maharashtra state (area 307,713 km^2^) has numerous large dams and its economy depends mostly on agriculture and industries. About 60% of the cultivable land of the state comes under the cultivation of cereal crops (1). Data on farmers were obtained from the Agricultural Census Division (33), while farmer suicide data were obtained from the various issues of Accidental Deaths and Suicides in India (ADSI) with the National Crime Records Bureau under the Government of India (15). Farmer population data are available once every 10 years (census), whereas the farmer suicide data are available annually. Therefore, to have similar time series, farmer population data were estimated for the other 4 years (from 2011 to 2014) through linear regression estimation between two data points. A linear equation [of the form *Y* = *mX* + *C*—where *Y* (farmer suicides) is the dependent variable and *X* (years) is the independent variable, while *m* is the slope and *C* is the intercept constant] was fitted between the annual total observed data to find the association and trend between farmer suicides and years. With the fitted equation, it was possible to understand the increasing trend and estimate the farmer suicides for years where data was missing. The annual farmer standardized suicide rate (per 100,000) were estimated from the number of farmer suicide each year divided by its population size of farmers.

Changes in TWS are premeditated worldwide at monthly resolution using GRACE as the satellite goes over each location monthly once. Scientists have authenticated the GRACE data in various publications previously [e.g., (29, 34–40)]. The land mass variations due to snow water equivalent were neglected in the analysis owing to Maharashtra's temperate climate. In fact, the monthly TWS data are available only as anomalies, as the GRACE team removes all the long term monthly averages during 2004–2009 from the actual data before providing the data for research. Thus, each TWS monthly grid indicates the deviation relative to the baseline average for Jan 2004–Dec 2009. GRACE data were downloaded data at a specific spatial (1°) and temporal- (monthly) resolutions. The Noah, a land surface model in GLDAS, uses remote sensing data to estimate soil moisture (SM) at similar spatio-temporal resolutions as GRACE data. The TWS and GLDAS-based soil moisture (SM) were used to determine GW storage anomalies as shown in other studies (28, 36, 39, 40):

(1) $$\begin{array}{r} \text{GW }=\text{ TWS}-\text{SM} \end{array}$$

where TWS, SM, and GW are all equivalent water thickness estimated in centimeters. This study aimed at identifying annual average GW trends, therefore monthly GRACE and GLDAS data were downloaded from 2002 to 2013 and averaged annually. Similar to the TWS, the monthly GW anomaly will represent the GW storage deviating from the baseline average for Jan 2004–Dec 2009.

India's Central Groundwater Board (CGWB) monitors GW levels in selected locations. As of 31 March, 2013 (41), it has maintained 1,302 monitoring wells in Maharashtra (1,075 dug wells and 227 piezometers at quarterly intervals (pre-monsoon or *Rabi* season in January; pre-monsoon in May; monsoon in August; post-monsoon or *Kharif* season in November). The government data can be accessed via online (www.india-wris.nrsc.gov.in), but the exact well-locations are not provided for scientific scrutiny. Without the exact location, elevation of wells cannot be known and thus the GW assessment becomes unfeasible. In addition to the CGWB depth to water data (WD), supplemental data on GW storage augmenting the CGWB data is needed (39). One such data is remote sensing data from GRACE.

Statistical Analysis System (SAS) software was used for analysis and mean values presented as ±1 standard deviation (42). The Pearson correlation method was also used to examine the relationships among number of farmers suicide, the standardized suicide rates (per 100,000), GW, GW 1 year lag, GW 2 years lag, WD, WD 1 year lag, and WD 2 years lag between 1996 and 2013. Then the analysis of variance (ANOVA) and regression analysis were further used to analyze those relationships between GW 2 years lag (X) and standardized suicide rates per 100,000 (Y) (42). Regression analysis was used to test relationships among standardized suicide rates (Y) and GW 2 years lag (X) between 2004 and 2013.

## Results and Discussions

**Figure 1:**
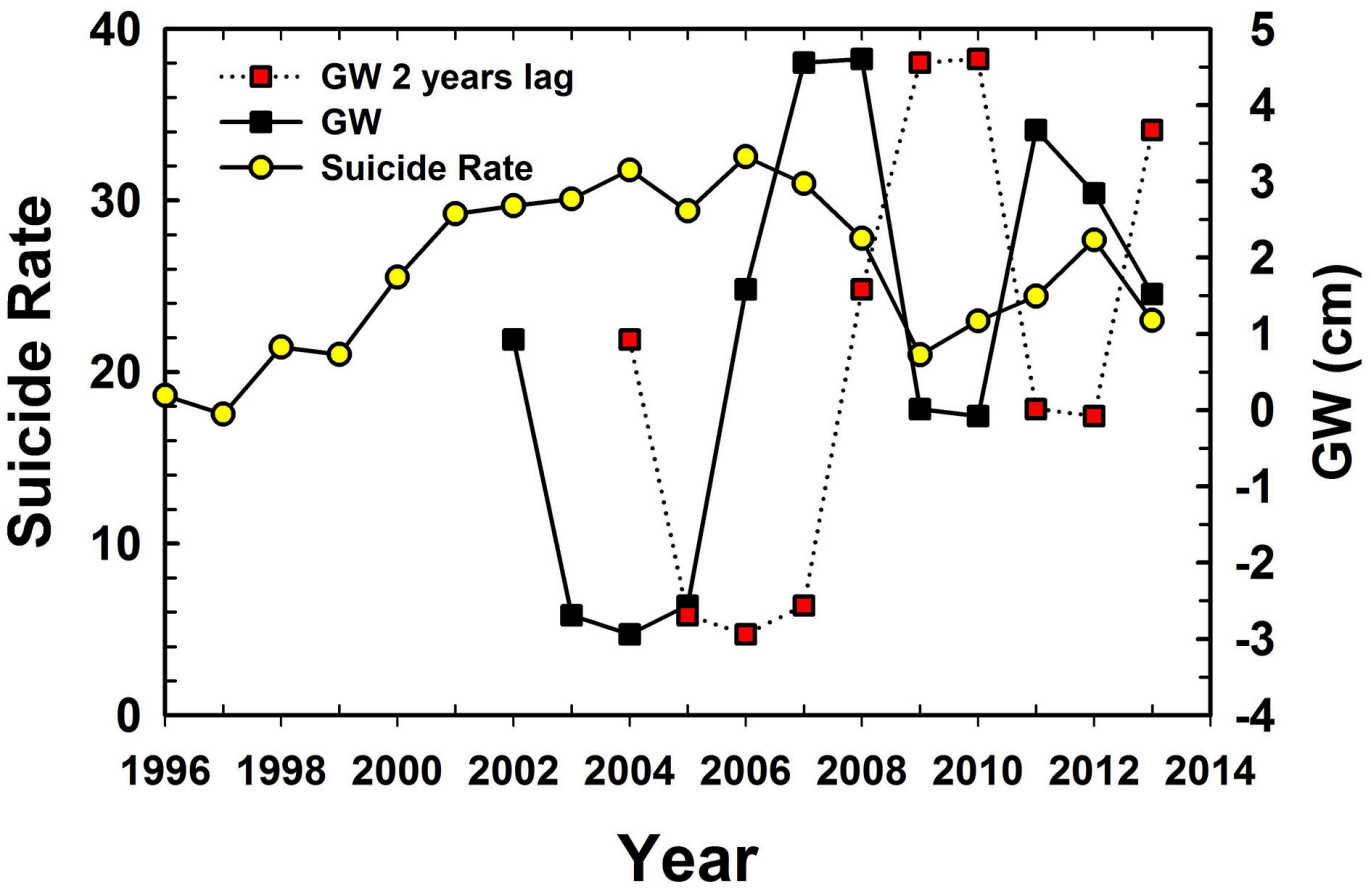
Comparisons among Maharashtra farmer suicide rate (per 100,000), annual GRACE estimated groundwater storage anomalies (GW) and GW 2 years lag.

The average number of farmers during the four census periods was 12.52 million (±1.46, *n* = 4; lowest 10.64 and highest 13.7 million). The farmer suicide cases (Figure 1) have increased from 1917 in 1997 to a maximum of 4,453 in 2006, and then decreased (15). The number of farmers also increased from 10.92 million in 1997 to 13.68 million in 2006, and then decreased to 13.67 million in 2011. Therefore, the standardized suicide rates (per 100,000) increased from 17.55 in 1997 by reaching a peak of 32.55 in 2006, then sharply decreased to 21.0 in 2009, and yet again increased to 27.7 in 2012 (Figure 1). The annual long-term average farmer suicide was 3,314.8 individuals (±760.3, *n* = 18, range 1,917–4,453) from 1996 to 2013, which equated to 9.1 farmer suicides per day in Maharashtra, India.

The GW storage anomaly fluctuated from −2.94 to 4.61 cm from 2002 to 2013 (Figure 1). The average anomaly was 0.95 cm (±2.71, *n* = 12), indicating an average trend that was above the baseline GW mean for 2004–2009. The least (−2.94 cm) and highest (4.61 cm) GW anomalies were noted in 2004 and 2008, respectively. The annual long-term depth to GW level (WD) from Gadchiroli CGWB well, a well in the most farmer suicide prone region of Vidarbha, showed an increase of 0.03 m during the study period. Thus, the GW levels have declined by 3 cm yearly from 1996 to 2012. The CGWB data on the WD for Gadchiroli had the least (2.94 m) and highest (5.80 m) values recorded in 2006 and 2010, respectively. The GW status for the well in Gadchiroli was therefore better in 2006 compared to 2010. However, the exact locations of wells were not known, hence the cause for the shallow water levels observed by CGWB could not be speculated.

The annual number of suicide by farmers and the standardized suicide rate were positively correlated (Pearson Correlation Coefficients *r* = 0.96, *p* < 0.001). The GW without lag was not significantly correlated with farmer suicide rate (*r* = −0.14, *p* > 0.66), while the GW with 1 year lag had a slightly better correlation (*r* = −0.55, *p* > 0.08; Table 1). This is due to the fact that the suicides mostly occurred during the first few months of the calendar year (mostly coinciding with non-monsoon months). Hence, the correlations are better when compared to antecedent GW storage conditions. The negative correlation indicates that as GW storage increases, the suicide decreases as there is more water available for preventing drought related suicide rates. Of the different correlations tested, the GW with 2 years lag had the highest correlation with the farmer suicide rates. The lag between actual monsoon season and peak groundwater recharge could be the reason for the observed higher correlations between GW and farmer suicide rates.

**Table 1:** Pearson correlation coefficients and test probability among number of farmers committed suicide, standardized suicide rate (per 100,000), GRACE derived groundwater storage (GW), CGWB groundwater level (WD), GW 2 years lag (with 2 years lag).

	**Standardized suicide rate**	**GW**	**WD**	**GW 2 years lag**
Farmer suicide	0.96***	−0.00	−0.17	−0.85**
Standardized suicide rate	1	−0.13	−0.22	−0.83**
GW		1	−0.34	−0.13
WD			1	0.69*
GW 2 y lag				1

*** *p < 0.001*,

** *p < 0.005*,

* *p < 0.05*.

**Figure 2:**
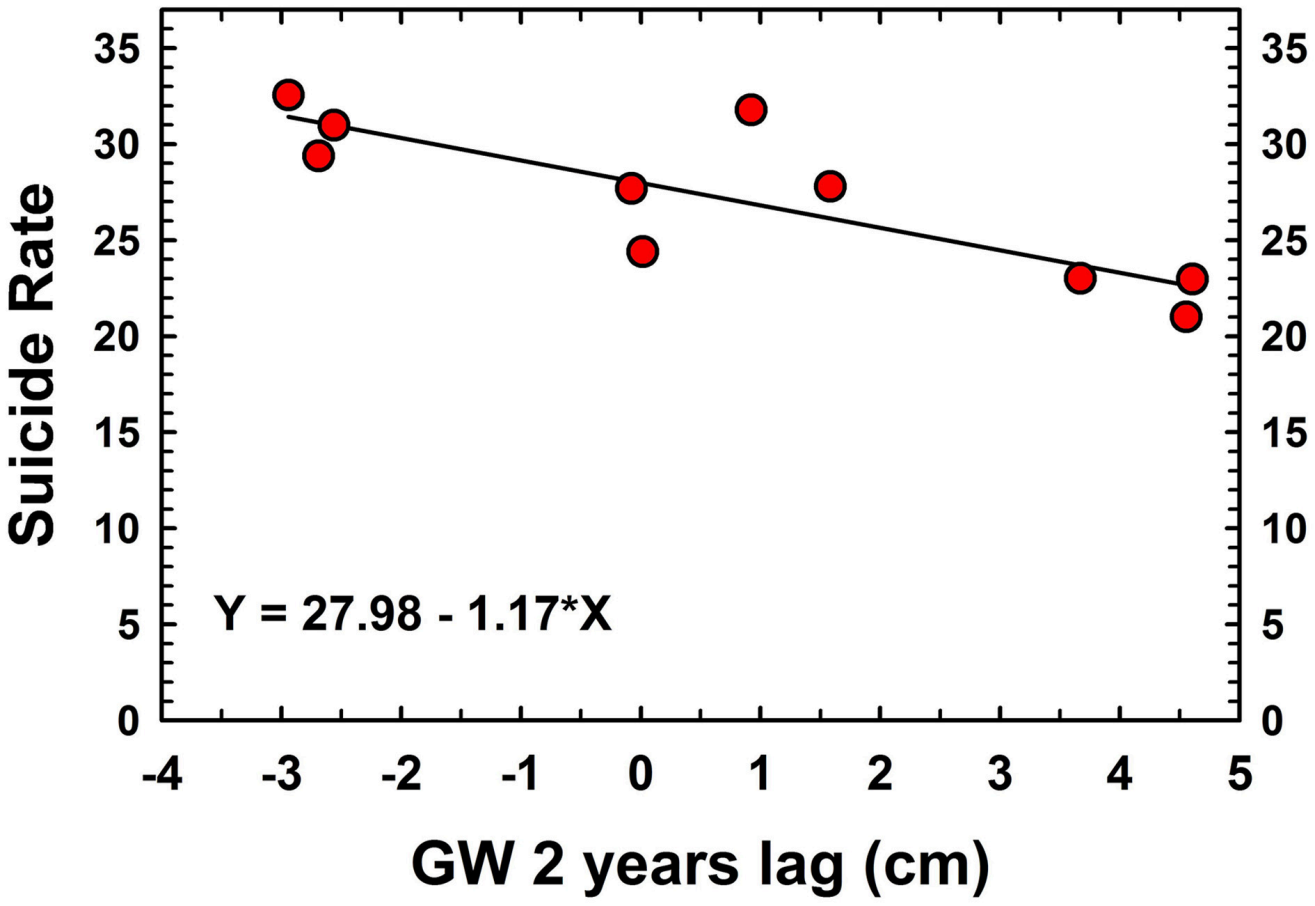
The regressions of Maharashtra farmer suicide rate (per 100,000, Y) in relation to 2 years lag of annual GRACE estimated groundwater storage anomalies (GW 2 years lag, X; solid regression line: *Y* = 27.98 – 1.17 *X, *R*^2^ = 0.69, *p* < 0.005).

The GW with 2 years lag was negatively correlated to farmers' standardized suicide rate (*r* = −0.83, *p* < 0.005) as well as with the number of suicide (*r* = −0.85, *p* < 0.005). In addition, GW 2 years lag was positively linked with CGWB depth (*r* = 0.69, *p* < 0.05; Figure 2). The GW with 2 years lag accounted 68.9% variations of farmer standardized suicide rate (*F*_1, 8_ = 17.72, *p* < 0.005). The regressions of farmer standardized suicide rate (per 100,000, Y) in relation to GW 2 years lag (cm, X) was: *Y* = 27.98 – 1.17 ^*^X, *p* < 0.005 (Figure 2). On the other hand, there were no significant correlations between the CGWB derived GW table level (WD) and farmer suicide rates (*r* = −0.21, *p* > 0.40), neither with 1 year lag (*r* = −0.24, *p* < 0.34) nor with 2 years lag (*r* = 0.18, *p* > 0.49; Figure 2). This clearly indicated issues regarding the location of the CGWB well and the limited spatio-temporal information captured due to less monitoring. Therefore, the GRACE estimated GW storage anomaly, with lag times can be used to recognize droughts and the knowledge derive from such findings has the potential to prevent future farmer suicide rates in Maharashtra and elsewhere in India.

Unlike the government-owned well data that are mostly restricted to unconfined aquifers, due to larger number of shallow well-monitoring stations (Central Ground Water Board or CGWB), the GRACE estimates can easily cover GW storage in both confined and unconfined areas by arriving at net GW storage changes covering large areas. In addition, the CGWB wells vastly vary depending on geological conditions and distance from pumping activity. So, the GRACE data have better prospective to provide an overall estimate of GW status across large areas including complete GW storage components and is more representative of the ground situation. This technology will therefore become essential in regions where deep bore wells induce stress to confined aquifers, as in the case of agriculture-reliant India. Hence, this study has focused on both confined and unconfined GW storage as estimated by GRACE and the results indicate strong correlation (*r* = −0.83, *p* < 0.005) between antecedent GW storage and farmer suicide rates for Maharashtra state.

About 40% of Vidarbha's farmers in Maharashtra wanted to quit farming due to farming stress, so more solution-focused investments, such as marketing, water storage infrastructures, rural credit, subsided bank loan, and self-help groups are needed (21). In 2005, the Mumbai High Court directed the Tata Institute of Social Sciences (TISS) to study the agrarian crisis in Maharashtra and the study found crop loss and inadequate irrigation facilities as the root-cause for the crisis (43). Later, the Indira Gandhi Institute of Development Research interviewed farmers (*n* = 116) across 109 villages and found that acute water issues, resulting from low rainfall and low recharge, were primarily affecting the crop output (18, 44). The central government's relief package included irrigation projects and construction of rainwater harvesting structures while the state government sanctioned USD 160 million through the Vidarbha Watershed Mission to decrease irrigation stress. But, the farmers were less satisfied with the relief package, as the timeliness of the relief packages were not adequate and compensation packages were not enough to cope with stress (45). So, it is clear from these reports that the issue of farmer suicide had been debated in literature and the primary causes include change in social status, harassment by loan sharks, mockery from community, economic loss, increasing farming cost, crop failure, drought, flood, water scarcity, health issues and alcohol abuse (17, 18, 20, 24).

In spite of various counter-measures to tackle the farmer suicide crisis, the GW storage component had been largely overlooked and underestimated so far. Groundwater levels are decreasing in Maharashtra in recent years while the pumping of GW increases in farmlands. Out of the total 76 taluks (“taluk” meaning subdivision of district with several villages) in the Marathwada region, the GW level dropped in 61 while in 25 taluks, the depletion drastically reached to 1–2 m during the last 5 years. Over 200,000 irrigation wells and 169,000 domestic wells have been installed and pumping water as of 2011 (46). Unsustainable access to GW for irrigation through the digging of deep bore wells has indeed pushed many farmers into serious economic debt, especially when the wells run out of water within a few years of installation. Sadly, farmers lack the hydrological understanding of the complex underground geology in the region where any attempt to drill to get water beyond 200 feet is impracticable. In addition, measures to enhance GW recharge are not generally practiced in the GW over-exploited zone, which indicates the enormity of the pressure on farmers who use groundwater that may of recharged many decades ago. This study shows significant statistical correlation between the actual water storage data and farmer suicide rate. So, the government can initiate rapid response as soon as the GW storage starts to decrease and this way, the crisis can be tackled timely. Such measures are already practiced in Australia (19, 24, 47).

Studies have showed strong correlations among droughts and farmer suicide rates in Australia where the government mobilized social workers and psychiatrists to provide immediate counteractive measures, thereby lessening the crisis (19, 47–49). Lessons learned from drought histories were used to convince farmers on choosing the best crop types and water saving measures, when predictions of drought/less than normal rainfall were available (47). This strategy can be adopted in India's farmer-suicide prone regions. Critiques however argue that the Indian suicide prevention measures are limited to political announcements on subsidies targeting the rural vote bank rather than proactive mitigation strategies (19). They argue that hands-on actions at the grassroots countering suicide should not be limited to government schemes aiming at counseling and subsidies. The measures must incorporate access to irrigation water, improvement in soil fertility and reversal of land degradation so that farmers can avoid future economic loss inflicted by crop failure.

India is a signatory to the United Nations Convention to Combat Desertification (UNCCD), so it has a mandate to neutralize land degradation by 2030 (50). But, it will be an uphill battle. Nevertheless, the construction of large number of decentralized irrigation structures, such as check dams, percolation tanks, and ponds, as well as renovating small dams, ponds and lakes to harvest rainwater have been successful to enhance GW storage and access in the increasingly degraded dryland regions of western India (51–54). The minor irrigation structures combined with sustainable management of soil, water and land resources are fundamental to reverse land degradation, restore GW recharge and enhance sustainable development in drylands (55). Such a model must be promoted uncompromisingly by the government as part of a national-level farmer-suicide mitigation strategy. The economic investments for land restoration and water infrastructure have been reported to enrich farmland soil quality, agricultural productivity, irrigation water availability and GW recharge in rural India (56–61).

It is important to involve specialists on water resource, irrigation and land restoration to identify suitable water conservation and land management methods to minimize water loss and land degradation (62, 63). Farmer counseling units must be deployed to provide therapy to depressed farmers while crop specialists can recommend better crops suitable for the terrain and fund delivery assisting them to invest in suitable farming tools. As reported in a study on the Australian agricultural vulnerability toward droughts and climate variability (64), the government should focus more on the execution of mitigation measures. Land and water managers must be fully prepared for the worst case scenarios of multiyear droughts due to uncertain future climate consequences. It's about time for the policy makers and politicians to shift their focus from the “re-active” to “pro-active” paradigm toward land and water linked issues involving the livelihoods of millions of Indian farmers. Prediction of farmer suicide scenarios, such as done with the model described in this study, can provide the government time to invest in small scale recharge structures and subsidy and counseling activities for farmers.

## Limitations

GRACE RL05 data grid size distribution is ~100 km at the study site Maharashtra, which is processed from an original data at 300–400 km resolution. As a result, only large scale areas can be studied using GRACE, and hence the study could not compare against other Indian states which have reported high farmer suicide rates (e.g., Punjab and Andhra Pradesh). In addition, GRACE data may contain errors due to signal leakages from neighboring grids, ocean mass grids (separate GRACE product), dominant geophysical features (e.g., mountains) and surface water bodies (65). To counter the signal leakage errors, a rescaling approach was employed, which was also discussed in the Methods section. The rescaling factors take in to account these errors, depending on location of the grid. This method is also reflected in many studies done globally [e.g., (28, 58)]. In regards to the surface water body mass changes and associated gravity anomaly, it was assumed that change in surface water levels are similar to the baseline average (average monthly surface water from 2004 to 2009), which was removed to arrive at anomalies. In addition, due to limitations in surface water data, and difficulties in obtaining these data from government agencies (which have classified them as sensitive data), it was not possible to use observation data. Hence, it was assumed that the removal of baseline average surface storage will result in net groundwater change (28, 58, 66).

Even though there are many factors that can lead to farmer suicide (27, 67), ranging from physical (e.g., water availability, climate change, etc.), economical (e.g., loss and low profit), mental health (23), and social issues, this study only focuses on the groundwater availability part, especially during the non-monsoon season (rabi) wherein groundwater is the major irrigation supply. In regards to mental health issues, the existence of depression, prior and current history of medical disorders, and cognitive impairment were reported to be the most important risk factors for suicides (23). While there have been studies that focused more on social, mental and economic aspects [e.g., (27)], studies on actual groundwater change and farmer suicide is absent. This study, for the first time, has aimed to bridge the gap in one specific area: groundwater availability—farmer suicide. The current study acknowledges the aforementioned range of issues, however, to keep more focus on the water availability theme, this study analyzed the relationship between groundwater availability and farmer suicide. Future studies are warranted in the other areas, especially social, climate change and economic issues, for which this current study can aid in developing a holistic framework.

## Conclusion

This paper presents evidence correlating the groundwater storage trend to farmer suicide. The correlation increases when the time lags are introduced and a 2 years lag has the highest correlation. Hence, GW storage rates could be used as an indicator mechanism to mitigate farmer suicide, at least 2 years in advance. This may increase awareness among land and water resource managers, policy-makers and politicians to formulate rapid counter measures to combat future farmer suicide incidences. Government of India, currently promotes the catchphrase “more crops per drop” to increase efficiency in land and water resource management. Moreover, the government plans to start satellite-based crop-monitoring that may help farmers to diversify crops and protect farmlands. So, it's about time to comprehend the importance of GW storage status to safeguard millions of farmers whose livelihoods depend on the humanity's most precious commodities: land and water.

## Author Contributions

PC and GA conceptualized the ideas for the paper, did the remote sensing analysis, and wrote the paper. MH did the statistical analysis for the paper.

### Conflict of Interest Statement

The authors declare that the research was conducted in the absence of any commercial or financial relationships that could be construed as a potential conflict of interest.
